# The Malaria Parasite Cyclin H Homolog PfCyc1 Is Required for Efficient Cytokinesis in Blood-Stage *Plasmodium falciparum*

**DOI:** 10.1128/mBio.00605-17

**Published:** 2017-06-13

**Authors:** Jonathan A. Robbins, Sabrina Absalon, Vincent A. Streva, Jeffrey D. Dvorin

**Affiliations:** a Division of Infectious Diseases, Boston Children’s Hospital, Boston, Massachusetts, USA; b Division of Infectious Diseases, Brigham and Women’s Hospital, Boston, Massachusetts, USA; c Department of Medicine, Harvard Medical School, Boston, Massachusetts, USA; d Department of Pediatrics, Harvard Medical School, Boston, Massachusetts, USA; University of Pittsburgh

**Keywords:** *Plasmodium falciparum*, asexual replication, cell cycle, malaria, schizogony

## Abstract

All well-studied eukaryotic cell cycles are driven by cyclins, which activate cyclin-dependent kinases (CDKs), and these protein kinase complexes are viable drug targets. The regulatory control of the *Plasmodium falciparum* cell division cycle remains poorly understood, and the roles of the various CDKs and cyclins remain unclear. The *P. falciparum* genome contains multiple CDKs, but surprisingly, it does not contain any sequence-identifiable G_1_-, S-, or M-phase cyclins. We demonstrate that *P. falciparum* Cyc1 (PfCyc1) complements a G_1_ cyclin-depleted *Saccharomyces cerevisiae* strain and confirm that other identified malaria parasite cyclins do not complement this strain. PfCyc1, which has the highest sequence similarity to the conserved cyclin H, cannot complement a temperature-sensitive yeast cyclin H mutant. Coimmunoprecipitation of PfCyc1 from *P. falciparum* parasites identifies PfMAT1 and PfMRK as specific interaction partners and does not identify PfPK5 or other CDKs. We then generate an endogenous conditional allele of PfCyc1 in blood-stage *P. falciparum* using a destabilization domain (DD) approach and find that PfCyc1 is essential for blood-stage proliferation. PfCyc1 knockdown does not impede nuclear division, but it prevents proper cytokinesis. Thus, we demonstrate that PfCyc1 has a functional divergence from bioinformatic predictions, suggesting that the malaria parasite cell division cycle has evolved to use evolutionarily conserved proteins in functionally novel ways.

## IMPORTANCE

Human infection by the eukaryotic parasite *Plasmodium falciparum* causes malaria. Most well-studied eukaryotic cell cycles are driven by cyclins, which activate cyclin-dependent kinases (CDKs) to promote essential cell division processes. Remarkably, there are no identifiable cyclins that are predicted to control the cell cycle in the malaria parasite genome. Thus, our knowledge regarding the basic mechanisms of the malaria parasite cell cycle remains unsatisfactory. We demonstrate that *P. falciparum* Cyc1 (PfCyc1), a transcriptional cyclin homolog, complements a cell cycle cyclin-deficient yeast strain but not a transcriptional cyclin-deficient strain. We show that PfCyc1 forms a complex in the parasite with PfMRK and the *P. falciparum* MAT1 homolog. PfCyc1 is essential and nonredundant in blood-stage *P. falciparum*. PfCyc1 knockdown causes a stage-specific arrest after nuclear division, demonstrating morphologically aberrant cytokinesis. This work demonstrates a conserved PfCyc1/PfMAT1/PfMRK complex in malaria and suggests that it functions as a schizont stage-specific regulator of the *P. falciparum* life cycle.

## OBSERVATION

Approximately half of the world’s population is at risk for malaria, with roughly 200 million infections and half a million deaths annually. There is now documented resistance to all clinically used antimalarial agents, making the specter of untreatable malaria a potential reality (1). Studying core biological processes of the organism may help elucidate novel pharmacological targets.

Blood-stage replication of *Plasmodium* species in humans is responsible for clinical malaria, and this replication entails an unusual cell division process called schizogony. Following invasion of a human erythrocyte, the newly invaded merozoite enters a growth period and then undergoes multiple asynchronous nuclear divisions within a common cytoplasm. To complete the division process, organelles are distributed to each nucleus, and membranes are partitioned to create new daughter merozoites. These daughter merozoites burst synchronously from the red blood cell to start the process anew by invading nearby erythrocytes. The asexual blood-stage is the major target for clinical treatment of *Plasmodium falciparum*.

Despite the critical importance of understanding genes involved in blood-stage proliferation for the development of new antimalarial therapies, remarkably little is known about cell cycle control in the malaria parasite. The *P*. *falciparum* core cell division apparatus appears poorly conserved compared to other eukaryotes based on genomic, bioinformatic, and limited experimental data (reviewed in reference 2).

Our core understanding of eukaryotic cell cycle control is based on the activity of complexes of cyclin and cyclin-dependent kinase (CDK), which phosphorylate substrates to promote cell cycle progression (reviewed in reference 3). This is best studied in yeast and animal cells, which collectively constitute a monophyletic branch of the eukaryotic tree and which are both very distantly related to *Plasmodium* spp. (4). CDK activity is generally turned on through the synthesis of cyclins, which bind to and activate CDKs. In the absence of cyclins, CDKs are not active. In model eukaryotes, before committing to a round of cell division, cells have low CDK activity. The onset of increased CDK activity commits cells to enter the cell cycle, progress through a G_1_ growth phase, undergo DNA replication (S phase), pass through a second G_2_ growth phase, and then enter into mitosis. The destruction of mitotic cyclins by the anaphase-promoting complex allows for exit from mitosis and completion of the cell cycle. This reestablishes a low-CDK state and allows the cell cycle to start anew.

Of note, there are also cyclin-CDK complexes whose primary roles do not involve cell cycle regulation. One important complex that falls into this category is the trimeric Cdk7/cyclin H/MAT1 complex (also known as transcription factor IIH [TFIIH]), which in yeast and metazoan cells is required for efficient transcription by RNA polymerase II. In some systems, Cdk7 also functions as a CDK-activating kinase, providing a link between transcription and cell cycle regulation. This function is less well conserved and is absent in yeast, for example (reviewed in reference 3).

At this time, it is difficult to reconcile the general understanding of the principles governing eukaryotic cell division with the blood-stage division process of the malaria parasite (reviewed in reference 2). It is of critical importance to understand this process as the proteins involved, particularly those that are divergent, would make appealing drug targets. Currently, the role of CDKs and cyclins in the *P. falciparum* replication cycle remains incompletely defined.

*Plasmodium* cyclins were initially identified from sequence homology and have been studied biochemically (5). There are, based upon a recent bioinformatic analysis, a total of three identifiable *Plasmodium falciparum* cyclins—PfCyc1 (PF3D7_1463700), PfCyc3 (PF3D7_0518400), and PfCyc4 (PF3D7_1304700). The initial annotation of PfCyc2 was erroneous (6). None of these cyclins cluster phylogenetically with the G_1_, S-phase, or mitotic cyclins of plants or animals (6). It is unknown whether they are involved in cell cycle progression in *P. falciparum*.

PfCyc1 aligns most closely with the cyclin H family of cyclins (5, 6), which in other organisms are responsible for promoting RNA polymerase II transcription as part of the trimeric cyclin H/Cdk7/MAT1 complex (7). However, PfCyc1 has been biochemically shown to be capable of activating both the Cdk1 homolog PK5 and the Cdk7 homolog MRK (8, 9). PfCyc1-MRK activity is further increased by the addition of MAT1 (9). Because these experiments were performed *in vitro* with recombinant protein, it remains unknown which of these interactions are physiologically relevant. Activation by PfCyc1 has also been demonstrated for a distantly related and nonessential CDK family kinase, PfCrk5 (10).

Of the remaining cyclins, PfCyc3 aligns most closely with plant P-type cyclins, and PfCyc4 aligns most closely with the cyclin L family (5, 6). The related *Plasmodium berghei* cyclin PbCyc3 has been demonstrated to be dispensable for blood-stage replication and is important (but not essential) for oocyst maturation in the mosquito host (6). No functional data exists for Cyc4 in any *Plasmodium* species.

Of the malaria parasite CDKs or CDK-like kinases, PfPK5 is the one highly conserved *P. falciparum* homolog of the canonical CDK Cdk1 (11). While Cdk1 is essential in yeast and mammals, deletion of PbPK5 (the PfPK5 homolog) in the mouse malaria *Plasmodium berghei* does not result in a detectable phenotype (12). It is unknown whether *P. falciparum* PK5 is essential or whether it is involved in cell division; a published attempt to disrupt it in blood-stage parasites was not successful, suggesting essentiality (13). From a structural standpoint, PfPK5 is similar to mammalian and yeast CDKs based on X-ray crystallography data (14). Biochemically, it retains a dependence on cyclins for kinase activity, and *in vitro*, it is promiscuously activated by the malaria parasite cyclins PfCyc1 and PfCyc3, as well as the mammalian cyclin A, cyclin H, p25, and the *Xenopus* RINGO (5, 8, 14). There are limited data regarding PfPK5 substrates. Notably, PfPK5 is reported to phosphorylate PfORC1, suggesting a role in in DNA replication (15).

Malaria parasites also contain a CDK, PfMRK, that shares the greatest sequence similarity with Cdk7. PfMRK interacts with PfMAT1 *in vitro*, and PfCyc1 stimulates PfMRK kinase activity (16). Surprisingly, PfMRK has been found to interact with DNA replication proteins by a bacterial two-hybrid assay (16). This suggests the possibility of nonconserved functions for this kinase complex. As PfPK5 does not require phosphorylation for activation *in vitro* (14), it is unlikely that PfMRK functions as a CDK-activating kinase for PfPK5.

Remarkably, no functional role for any cyclin has yet been described in blood-stage replication of any *Plasmodium* species. This project was undertaken to functionally identify *Plasmodium* proteins involved in cell cycle progression using complementation in budding yeast and to begin to clarify cyclin-CDK pairings within the malaria parasite. In addition, we demonstrate an essential role for PfCyc1 during the asexual blood stage of *P. falciparum*.

### PfCyc1 complements yeast G_1_ cyclins.

**FIG 1:**
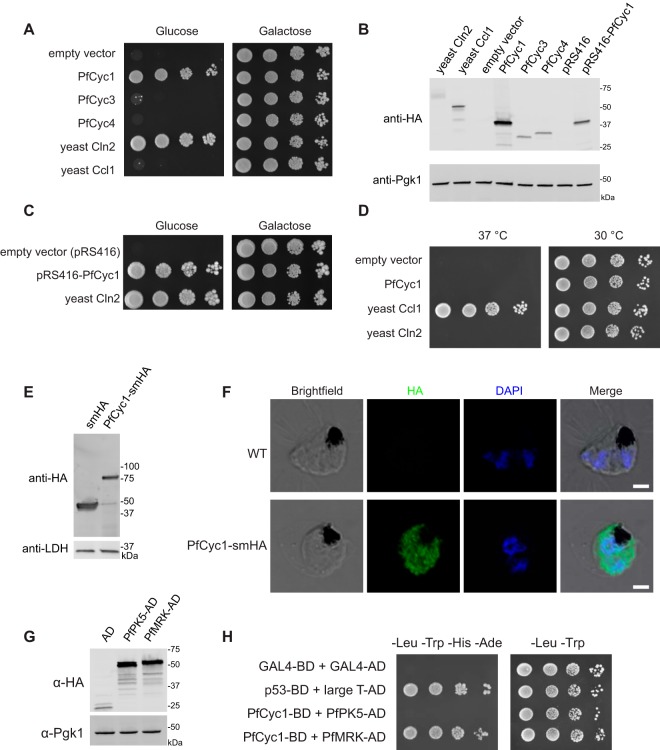
PfCyc1 complements yeast G_1_ cyclins. (A) Tenfold serial dilutions of *cln1*, *cln2*, *cln3 GAL1-CLN3* strains transformed with the indicated plasmids and plated on synthetic complete medium lacking uracil with either glucose (left) or galactose (right) as a carbon source. All cyclins contain a C-terminal tandem HA epitope. (B) Immunoblots against the strains in panels A and C. (C) Tenfold serial dilutions as in panel A with the indicated plasmids. pRS416-PfCyc1 expression has been placed under the control of a truncated promoter in a single-copy plasmid to reduce protein expression level. (D) A *ccl1-ts* strain was transformed with the indicated plasmids, and 10-fold serial dilutions were plated onto synthetic complete medium lacking uracil but with glucose and grown at either 30°C (permissive) or 37°C (restrictive). (E) Immunoblots of lysates from transgenic *P. falciparum* expressing either smHA or PfCyc1-smHA under the control of the Pfhsp86 promoter. (F) Indirect immunofluorescence staining with antibodies against the HA epitope (green) with DAPI counterstaining (blue) on PfCyc1-smHA-expressing parasites or wild-type (WT) controls. Bars, 2 µm. (G) Immunoblot against HA epitope present on PfPK5-AD (activation domain) and PfMRK-AD hybrid proteins to confirm protein expression in yeast. α-HA, anti-HA. (H) Tenfold dilutions of yeast two-hybrid reporter strains containing the indicated bait and prey plasmids. Plating onto synthetic complete medium lacking leucine and tryptophan (–Leu –Trp) selects for the bait and prey plasmids, and plating onto medium lacking leucine, tryptophan, histidine, and adenine (–Leu –Trp –His –Ade) additionally selects for physical interaction of the bait and prey fusion proteins. p53-AD and large T-AD are positive-control plasmids.

Given the lack of identifiable G_1_, S, or mitotic cyclins in the malaria parasite genome, we specifically tested whether bioinformatically identifiable malaria parasite cyclins are capable of functioning as cell cycle cyclins. We codon optimized *P. falciparum* cyclins with a tandem hemagglutinin (HA) tag and placed them in a yeast strain with its endogenous G_1_ cyclins deleted (*cln1*Δ *cln2*Δ *cln3*Δ) and a copy of *CLN3* placed under the control of a galactose-inducible promoter. This strain can grow on galactose but not on glucose. This complementation strategy has been used extensively for the identification of G_1_, S, and mitotic cyclins from multiple organisms. PfCyc1 complements yeast G_1_ cyclins, while PfCyc3 and PfCyc4 do not (Fig. 1A). Expression for all plasmids was confirmed by immunoblotting (Fig. 1B).

Of note, the expression level of PfCyc1 was significantly higher than for PfCyc3, PfCyc4, Cln3, or Ccl1. This raised the possibility that complementation was secondary to the higher protein concentration rather than intrinsic protein characteristics. To lower the expression level of PfCyc1 in this system, we truncated the ADH1 promoter and placed the allele into a CEN/ARS plasmid, which is typically maintained as a single copy (versus the 50+ copies expected from a 2µm plasmid). This reduces both the number of available gene copies to transcribe from, and the overall transcription level from each copy. This construct results in reduced protein expression level and remains able to complement yeast G_1_ cyclins (Fig. 1B and C).

### PfCyc1 fails to complement *CCL1*, the *S. cerevisiae* cyclin H homolog.

PfCyc1 has highest homology to cyclin H (5), which is required for efficient RNA polymerase II transcription in budding yeast and other eukaryotes. In some organisms, cyclin H is additionally responsible for activation of Cdk1 (17, 18), although this is not present for the budding yeast Cdk1 *Cdc28* (19) or for the malaria Cdk1 PfPK5 (14). To test whether PfCyc1 complements cyclin H deficiency, a high-copy-number plasmid expressing PfCyc1 was placed in a yeast strain harboring the temperature-sensitive *ccl1-ts4* allele of the essential cyclin H homolog *CCL1*. It has previously been demonstrated that human cyclin H can complement yeast *CCL1* (20). We confirmed expression of these plasmids and found that PfCyc1 was not capable of rescuing viability at the restrictive temperature (Fig. 1D).

### PfCyc1 interacts specifically with PfMRK in *P. falciparum*.

Given the unexpected behavior of PfCyc1 in the heterologous budding yeast system, we sought to study PfCyc1 in malaria parasites. An episomal construct coding for the full-length PfCyc1 fused at the 3′ end to spaghetti monster HA (smHA) was constructed. This smHA protein is composed of multiple HA epitopes incorporated into the backbone of a dark green fluorescent protein (GFP), allowing for higher avidity interaction due to the multiple copies of the epitope (21). Immunoblotting against whole-parasite extracts for PfCyc1-smHA revealed a dominant band of the expected size (Fig. 1E); the smHA control ran slightly slower than expected (band at ~46 kDa; calculated mass of 39 kDa). Indirect immunofluorescence against the HA epitope in parasites ectopically expressing PfCyc1-smHA revealed diffuse staining (Fig. 1F). The presence of cytoplasmic PfCyc1 in the parasite suggests a transcription-independent function for the protein.

PfCyc1 has previously been reported to activate multiple malaria CDKs, including PfPK5, PfMRK, and PfCrk5, by *in vitro* kinase assays with recombinant proteins (8–10). To clarify cyclin-CDK pairings in malaria, we performed an unbiased coimmunoprecipitation of episomally expressed smHA-tagged PfCyc1. Biological duplicate coimmunoprecipitations were performed and subjected to mass spectrometry. For a control, smHA alone was expressed from the same plasmid (expression confirmed in Fig. 1E). To filter for strongly reproducible interactions, we identified all proteins that were exclusively in the PfCyc1 coimmunoprecipitation (absent from the smHA control) and were represented by more than two peptides in both biological replicates; these proteins and their peptide counts are listed in Table 1. PfMAT1 and PfMRK were recovered specifically as interacting with PfCyc1. Notably, no other CDKs were present in either of the coimmunoprecipitation experiments, even without the selection criteria noted above (all data given in Table S1 in the supplemental material).

10.1128/mBio.00605-17.4 TABLE S1  Complete results from coimmunoprecipitation and mass spectrometry. Download TABLE S1, XLSX file, 0.1 MB. Copyright © 2017 Robbins et al. 2017 Robbins et al. This content is distributed under the terms of the Creative Commons Attribution 4.0 International license.

**TABLE 1:** Proteins identified by PfCyc1-smHA coimmunoprecipitation followed by unbiased mass spectrometry^a^

Protein	Expt 1		Expt 2		No. of total peptides in:	
	No. of unique peptides	No. of total peptides	No. of unique peptides	No. of total peptides	Control no. 1	Control no. 2
PfCyc1	22	52	17	23	0	0
PfMAT1	19	22	16	17	0	0
PfMRK	11	11	9	9	0	0

^a^ All proteins unique to PfCyc1-smHA coimmunoprecipitation represented by more than two peptides on both biological replicates are listed.

### PfCyc1 interacts specifically with PfMRK in a yeast two-hybrid assay.

To test the pairwise interaction of PfCyc1 with the CDKs PfMRK and PfPK5, we performed a yeast two-hybrid assay using PfCyc1 as bait. Expression was confirmed for both PfPK5 and PfMRK (Fig. 1G). It is noteworthy that PfMRK required codon optimization for efficient expression, while the cDNA sequence of PfPK5 was expressed efficiently without codon optimization. We found that PfCyc1 interacts with PfMRK but not PfPK5 in this assay (Fig. 1H). Together with the mass spectrometry data, these findings suggest the presence of a conserved PfCyc1/PfMAT1/PfMRK trimeric complex in live *P. falciparum* parasites.

### Construction of a conditional PfCyc1 allele in blood-stage *P. falciparum*.

**FIG 2:**
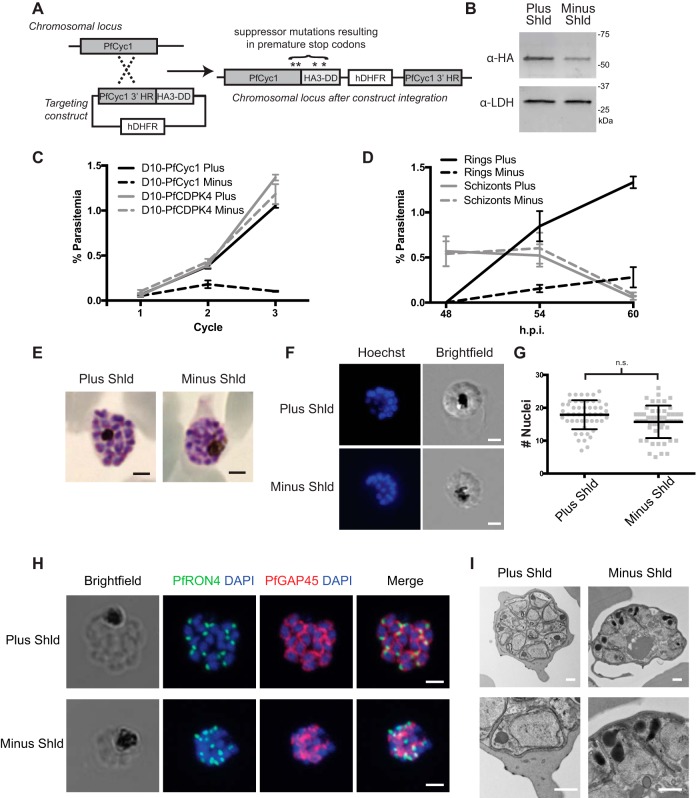
PfCyc1 is critical for blood-stage parasites. (A) Schematic of single-crossover vectors to generate D10-PfCyc1-DD parasites (not drawn to scale). The endogenous locus following homologous recombination is shown on the right. Asterisks above indicate the locations of selected suppressor mutations. HR, homology region; hDHFR, human dihydrofolate reductase. (B) Immunoblot of protein lysates from D10-PfCyc1-DD schizont-stage parasites cultured in the presence (Plus) and absence (Minus) of Shield-1 (Shld) and probed with antibodies to HA or PfLDH (loading control). (C) Replication curves of D10-PfCyc1-DD and PfCDPK4-DD parasites cultured in the presence and absence of Shld (*n* = 3; mean ± standard deviation [SD]). (D) Absolute schizont-stage and ring-stage parasitemia from D10-PfCyc1-DD parasites cultured in the presence and absence of Shld at 48, 54, and 60 h postinvasion (h.p.i.) (*n* = 3; mean ± SD). (E) Field’s stain of E-64-treated D10-PfCyc1-DD schizonts cultured in the presence and absence of Shld. (F) Fluorescence microscopy of live parasites stained with Hoechst 33342. Bars, 2 µm. (G) Quantification of the number of individual nuclei from live E-64-treated schizonts cultured in the presence and absence of Shld (*n* = 50; mean ± SD). n.s., not significantly different. (H) IFA of parasites grown in the presence and absence of Shld probed with antibodies against PfRON4 (green) and PfGAP45 (red). Bars, 2 µm. (I) Transmission electron microscopy of schizonts grown in the presence and absence of Shld and treated with E-64 50 h postinfection. Bars, 500 nm (for both top and bottom panels in panel I).

To study the function of PfCyc1 in living parasites, we generated transgenic parasites where the endogenous allele of PfCyc1 is fused to a destabilization domain (DD) and three copies of the HA epitope tag through a 3′ crossover approach (Fig. 2A). Parasites were subcloned, and the genotype was confirmed by whole-genome sequencing. In the absence of the stabilizing compound Shield-1 (Shld), PfCyc1-DD was degraded, resulting in a 60% ± 10% reduction in the steady-state protein level at 24 h postinvasion (Fig. 2B).

### PfCyc1 is required for blood-stage replication.

Sorbitol-synchronized ring-stage parasites were washed and then released into media either with or without Shld to test the effect of PfCyc1 depletion on parasite growth. For a control, the nonessential gene PfCDPK4 with the same DD tag was used. We found that D10-PfCyc1-DD parasites fail to proliferate in the absence of Shld (Fig. 2C). To further test the essentiality of PfCyc1, we performed a spontaneous suppressor screen in these parasites by growing separate clones in the absence of Shld but maintaining WR99210 selection. When wild-type growth returned, four independent lines were subjected to whole-genome sequencing. Analysis revealed spontaneous intragenic suppressors with mutations within PfCyc1-DD itself. All sequenced mutations either resulted in premature stop codons just prior to the DD or within the DD itself (Fig. 2A and Table S2). No other mutations were identified in the sequenced genomes.

10.1128/mBio.00605-17.5 TABLE S2  Locations of suppressor mutations. Download TABLE S2, XLSX file, 0.02 MB. Copyright © 2017 Robbins et al. 2017 Robbins et al. This content is distributed under the terms of the Creative Commons Attribution 4.0 International license.

### PfCyc1 is present throughout the cell cycle.

Given that cyclins in other systems promote cell cycle progression through oscillations in their own protein levels, we sought to determine whether PfCyc1 is present throughout the cell cycle. Synchronized PfCyc1-DD parasites grown in the presence of Shld were harvested every 12 h and immunoblotted against the HA epitope (Fig. S1). PfCyc1 was readily detected throughout the cell cycle, in contrast to the marked oscillations seen with cell cycle type cyclins in other characterized systems.

10.1128/mBio.00605-17.1 FIG S1  Immunoblot of PfCyc1-DD throughout asexual development. Parasite lysates were harvested every 12 h and immunoblotted with antibodies against HA (recognizes triple HA epitope within PfCyc1-DD) and PfLDH (loading control). Protein loading was normalized by PfLDH expression. Download FIG S1, EPS file, 2.5 MB. Copyright © 2017 Robbins et al. 2017 Robbins et al. This content is distributed under the terms of the Creative Commons Attribution 4.0 International license.

### PfCyc1 is required for progression through schizogony.

To determine whether PfCyc1 has a stage-specific activity, parasites were tightly synchronized and released in the presence or absence of Shld. Depletion of PfCyc1 did not alter progression from rings to trophozoites or from trophozoites to schizonts (Fig. S2). At 48 h postinvasion, the number of schizonts was the same with or without Shld. However, 6 h later, physiological egress in parasites with Shld produced more rings (0.85 ± 0.17%) than the parasites without Shld parasites (0.16% ± 0.04%). The absolute difference was stronger after an additional 6 h with 1.3% ± 0.07% and 0.28% ± 0.11% in parasite cultures with and without Shld, respectively (Fig. 2D). We note that while the life cycle of the parasites was prolonged due to the frequent manipulation required for the assay, the duration of the life cycle was similar in the presence and absence of Shld (Fig. S2). We observed that the PfCyc1-deficient schizonts appeared abnormal late in the time course. To directly evaluate the morphology of the transgenic schizonts, we examined E-64-treated schizonts 54 h postinvasion. E-64 is a cysteine protease inhibitor that prevents schizont rupture while allowing the development of viable merozoites. Field’s staining of PfCyc1 knockdown parasites at 54 h postinvasion revealed multinucleated schizonts with tightly packed nuclei, suggesting incomplete segmentation of daughter merozoites (Fig. 2E).

10.1128/mBio.00605-17.2 FIG S2  Stage progression throughout asexual development. D10-PfCyc1-DD parasites were tightly synchronized by magnetic purification of schizonts followed by sorbitol synchronization of newly invaded rings after 4 h in three replicates. Thin smears were obtained every 6 h. A total of 100 parasites were counted from each smear and scored as ring, trophozoite, or schizont. The percentage of each stage is relative and does not reflect the total parasitemia. Download FIG S2, EPS file, 1.5 MB. Copyright © 2017 Robbins et al. 2017 Robbins et al. This content is distributed under the terms of the Creative Commons Attribution 4.0 International license.

### PfCyc1 knockdown does not affect nuclear division.

The abnormal Field’s stained schizonts suggested one of two (nonmutually exclusive) possibilities: either there was a defect in the division of the nuclei themselves, or there was a defect in the segmentation process that separates these nuclei from each other into daughter merozoites. The methanol fixation step of Field’s staining dehydrates cells, and there is the possibility that distinct nuclei would not be resolvable from one another if the intervening structures separating nuclei (such as membranes or inner membrane complex) are aberrant. To clarify this and characterize the nuclei in PfCyc1 knockdown cells, synchronized live parasites were treated with E-64, stained with Hoechst 33342, sectioned, and imaged in serial sections. This revealed unremarkable nuclei in expected numbers per schizont in both the presence and absence of Shld (Fig. 2F and G).

### PfCyc1 is required for cytokinesis.

To better characterize the segmentation in PfCyc1-depleted malaria parasites, immunofluorescence assays (IFAs) were performed on synchronized E-64-blocked cells grown either in the presence or absence of Shld. Staining for PfGAP45, a soluble protein that complexes with PfGAP50 (along with PfMyoA and PfMTIP) to localize to the inner membrane complex (IMC) (22), revealed readily detected expression of the PfGAP45 protein but the absence of normal morphology in PfCyc1-deficient parasites (Fig. 2H). In contrast, IFAs against the rhoptry protein PfRON4 suggests unencumbered rhoptry formation. Similarly, IFA against the merozoite surface protein PfMSP1, which localized to the plasma membrane, demonstrated a failure to segment normally (Fig. S3).

10.1128/mBio.00605-17.3 FIG S3  Immunofluorescence of PfMSP1 in D10-PfCyc1-DD parasites. Parasites were grown with or without Shld, treated with E-64 as schizonts, and harvested. IFA was performed with antibodies against PfMSP1. Download FIG S3, EPS file, 33.7 MB. Copyright © 2017 Robbins et al. 2017 Robbins et al. This content is distributed under the terms of the Creative Commons Attribution 4.0 International license.

To better understand what structures’ formation depends on PfCyc1, we performed transmission electron microscopy on synchronized parasites. While no differences were noted in rings to early stage schizonts, late E-64-arrested schizonts demonstrated abnormal architecture of developing merozoites (Fig. 2I). Morphologically normal rhoptries are visible at the apical end, and nuclei are unremarkable. However, enclosure of the nascent merozoite itself fails, leaving the basal aspect of the merozoites disordered and the merozoite cytoplasm remaining topologically contiguous with the common cytoplasm.

### Conclusions.

The central nature of cyclin-CDK complexes in cell cycle control has been demonstrated throughout the eukaryotic kingdom, including in fungi, metazoans, plants, and early branching eukaryotes such as *Giardia*. The lack of identifiable G_1_, S, or mitotic cyclins in malaria-causing *Plasmodium* species is striking. This absence is most likely the result of secondary loss, as B-type cyclins are present in other members of the Apicomplexa phylum such as *Cryptosporidium* species, as well as in the related free-living flagellates *Chromera velia* and *Vitrella brassicaformis* (23).

The initial goal of this study was to identify *Plasmodium* genes capable of activating a cell cycle CDK in a heterologous system, and this revealed the putative cyclin H homolog PfCyc1. This is the first time that a *Plasmodium* cyclin has been demonstrated to be functional in a heterologous system. Notably, the *Toxoplasma gondii* cyclin TgCyc1 has been reported to have the same ability (24), although its function in *Toxoplasma* remains unknown. Interestingly, TgCyc1 also shares sequence similarity with cyclin H, although it is ~65% larger with numerous sequence insertions internally and sequence extensions at both N and C termini.

PfCyc1 was not able to complement the yeast cyclin H homolog *CCL1*, despite the bioinformatically predicted orthology. Of note, human cyclin H has previously been reported to complement yeast *CCL1* (20), indicating that the function of Ccl1 is not specific to yeast. Taken together, this finding suggests that PfCyc1 may have divergently evolved in apicomplexan parasites.

There is precedent for functional plasticity of cyclin H-containing complexes in other organisms. In metazoans, the cyclin H/Cdk7/MAT1 complex functions as a CDK-activating complex, while this role is absent in the budding yeast orthologous complex of Ccl1/Kin28/Tbf3 (17–19). It is possible that the apicomplexan orthologous complex has evolved distinct substrate specificity. It is unknown whether PfCyc1 is involved in transcription, as would be expected from bioinformatic analyses. The relative abundance of PfCyc1 in the cytoplasm suggests the possibility of a nontranscriptional function. While at this time we cannot formally exclude the possibility that this localization is an artifact of overexpression, we have found the same vector expressing a nuclear localization signal (NLS)-GFP fusion (25) to result in exclusively nuclear localization. We were unable to detect the endogenously tagged PfCyc1 allele we constructed by IFA despite attempts with multiple antibodies and fixation protocols.

Consistent with bioinformatic predictions, coimmunoprecipitation of PfCyc1 demonstrated that the PfCyc1/PfMAT1/PfMRK trimeric complex is preserved in malaria parasites, and is similar in constituents to the cyclin H/MAT1/Cdk7 complex. Individual pairwise interactions have been demonstrated previously (16). However, this is the first biochemical identification of the trimeric complex of PfCyc1/PfMAT1/PfMRK in an apicomplexan species, and the first isolation of this complex from live parasites. Importantly, this finding suggests that PfCyc1 serves as an *in vivo* activator of PfMRK alone, rather than the promiscuous cyclin-CDK pairings reported from *in vitro* biochemical studies. This clarifies our understanding of reconstituted biochemical experiments, which have demonstrated PfCyc1 to be a promiscuous CDK activator *in vitro* in the setting of likely supraphysiological concentrations of both PfCyc1 and CDKs.

One potential caveat is that, as the PfCyc1/PfMAT1/PfMRK complex was coimmunoprecipitated from late schizonts, it is formally possible that PfCyc1 interacts with other CDKs or other proteins at other stages of the cell cycle. Importantly, PfPK5 and PfCrk5 are both present in schizonts (10, 26), so one cannot ascribe lack of identified interaction to an absence of protein. It is also formally possible that PfCyc1 has weak, transient interactions with CDKs such as PfPK5 that are biologically significant but would not be identified in this experiment.

One intriguing possibility raised by the coimmunoprecipitation results is that the physiological activators of PfPK5 and PfCrk5 may not be cyclins and may be other unidentified proteins. Noncyclin CDK activators are well described in other systems, including p25 and RINGO/Speedy. It is unlikely that malaria noncyclin CDK activators would be identifiable based solely upon genome sequence data.

Functional genetic studies of PfCyc1 revealed a stage-specific requirement for PfCyc1 for proper cytokinesis. PfCyc1 depletion did not interfere with nuclear division. Given that this is a knockdown, it is possible that there are level-dependent activities of PfCyc1; i.e., that the residual levels of PfCyc1 are required for and sufficient to proceed past other earlier cell cycle steps. It has been proposed for other systems that increasing levels of activity order cell cycle progression, and this knockdown could conceivably block activity at a level above that required for nuclear division but below that required for cytokinesis.

Future studies will be informative in elaborating the mechanism by which PfCyc1 regulates cytokinesis in the parasite. The stage-specific block in segmentation seen in both the first and second cycles argues against a nonspecific transcriptional block, as a pure transcriptional block would lead to variable stage arrests based upon stochastic deprivation of essential proteins—or likely arrest as trophozoites if synchronized as rings, as for PfRACK1 (27). Additionally, the PfCyc1 knockdown parasites retain the ability to synthesize essential RNA polymerase II transcripts such as histones, as well as construct novel protein-containing organelles, including rhoptries.

There are several mechanistic possibilities for PfCyc1 function, including direct phosphorylation of substrates involved in cytokinesis and regulation of specific transcripts required for cytokinesis (either transcriptionally or translationally). Our future studies will work to address these questions and further clarify the mechanistic control of cytokinesis by this cyclin.

In conclusion, this work establishes an unanticipated function for PfCyc1 in a heterologous system and clarifies the biochemical composition of the PfCyc1-containing complex in *P. falciparum*. Taken together, these findings suggest that PfCyc1 has an essential and remarkable specific function in the segmentation process of blood-stage *P. falciparum* parasites.

### Methods. (i) Yeast G_1_ cyclin complementation.

For complementation testing, the *Saccharomyces cerevisiae* strain 1607-5D (*MAT*α *cln1*Δ* cln2*Δ* cln3*Δ* leu2*::*LEU2*::*GAL1*::*CLN3*) was transformed by standard methods with the indicated plasmid, and 10-fold serial dilutions were performed on plates containing synthetic complete medium minus uracil with either glucose or galactose as a carbon source.

### (ii) Plasmids.

PfCyc1, PfCyc3, and PfCyc4 were ordered as GeneArt Strings (Invitrogen), PCR amplified, and cloned into pBEVY-U using primers and restriction sites noted in Table S3 in the supplemental material. Yeast two-hybrid plasmids were cloned as indicated in Table S3. Spaghetti monster (21) episomal plasmids were constructed using primers in Table S3. PfCyc1 was PCR amplified and cloned into an episomal expression plasmid using NotI and XhoI. smHA was PCR amplified from pCAG_smFP HA (catalog no. 59759; Addgene) and cloned at the 3′ end with XhoI and KpnI to create pJR118. To construct a control plasmid consisting of the smHA tag alone, pJR118 was cut with NotI and XhoI, Klenow blunted, and self-ligated to excise PfCyc1 to create pJR152. To generate the PfCyc1-DD single-crossover construct, the 3′ fragment of PfCyc1 was PCR amplified using primers in Table S3 and cloned into the HA3-DD-containing plasmid pJR05 using NotI and XhoI.

10.1128/mBio.00605-17.6 TABLE S3  Sequences of primers and synthesized genes. Download TABLE S3, XLSX file, 0.02 MB. Copyright © 2017 Robbins et al. 2017 Robbins et al. This content is distributed under the terms of the Creative Commons Attribution 4.0 International license.

### (iii) Western blot analysis.

*S. cerevisiae* protein lysates were prepared from 5-ml portions of log-phase culture. The yeast cells were pelleted, resuspended in yeast protein extraction buffer (10 mM Tris base, 0.6% SDS) with protease inhibitors (phenylmethylsulfonyl fluoride [PMSF], aprotinin, leupeptin, pepstatin A, and tetrasodium phosphate), and mechanically disrupted by vortexing with glass beads, and sample buffer with beta-mercaptoethanol was added to the cells. *P. falciparum* lysates were obtained by lysing infected erythrocytes in 0.2% saponin in phosphate-buffered saline (PBS) with protease inhibitors (catalog no. S8820; Sigma) for 2 min, washing in PBS with protease inhibitors to clear hemoglobin, and resuspending the resulting parasite pellets in Laemmli buffer with beta-mercaptoethanol. Proteins were separated on mini-Protean TGX gels (Bio-Rad) and transferred to nitrocellulose membranes with a Trans-blot Turbo apparatus at 25 V and 1.3 A for 7 min. Blocking and antibody probing were performed in Odyssey blocking buffer diluted 1:5 in PBS. The primary antibodies mouse anti-HA at 1:1,000 (clone 2-2.2.14 [catalog no. 26183; Pierce]), mouse anti-Pgk1 at 1:10,000 (Invitrogen), and mouse anti-PfLDH (anti-*P*. *falciparum* lactate dehydrogenase) (1:1,000) were used to probe blots, and 800CW donkey anti-mouse near-infrared secondary (LiCor) was used for primary antibody detection. Imaging and volumetric quantification were performed using ImageStudio software and a LiCor Odyssey CLx imager.

### (iv) Transgenic *Plasmodium falciparum* strains.

The 3D7 strain of *P. falciparum* was maintained by standard methods as previously described (28) at 4% hematocrit. Sorbitol-synchronized ring-stage parasites at 2% parasitemia were transfected with ~100 μg plasmid DNA by electroporation and selected for with WR99210 (Jacobus Pharmaceutical Company). For coimmunoprecipitation studies, parasites (200 ml; 4% hematocrit parasites at ~5% parasitemia) were synchronized with sorbitol and collected as schizonts 36 to 48 h postinfection, and erythrocytes were lysed in 0.2% saponin in PBS with EDTA-free protease inhibitors (catalog no. S8830; Sigma). Free parasites were lysed in 0.5% Triton X-100 in PBS with EDTA-free protease inhibitors, diluted 1:2 into Tris-buffered saline with 0.05% Tween (TBST), and immunoprecipitated with anti-HA magnetic beads (catalog no. 88836; Pierce) at room temperature for 30 min. The beads were washed four times with TBST, and coimmunoprecipitates were collected by boiling for 5 min in 1× sample buffer with 4% beta-mercaptoethanol. The samples were run on 10% SDS-polyacrylamide gels and stained with 0.1% Coomassie blue R250, and the entire lane was cut and submitted for liquid chromatography coupled to tandem mass spectrometry (LC/MS/MS) analysis.

Immunofluorescence microscopy was performed on dried blood smears fixed with 4% paraformaldehyde, permeabilized with 0.1% Triton X-100 for 10 min, and blocked with 3% bovine serum albumin (BSA) for 1 h at room temperature. Rat anti-HA (clone 3F10; Roche; 1:50), mouse anti-PfRON4 (1:200), rabbit anti-PfGAP45 (1:5,000), and/or mouse anti-PfMSP1 (clone 1E1; 1:500) was used for 1 h and washed three times in PBS, and Alexa Fluor 488 or 555 secondary antibodies were applied (1:1,000; Molecular Probes). Slides were again washed three times in PBS and mounted with Vectashield containing 4′,6′-diamidino-2-phenylindole (DAPI) (Vector Laboratories). Images were obtained with a Zeiss LSM700 laser-scanning confocal microscope using a 63× 1.4-numerical aperture objective using Zen black software (Carl Zeiss, Inc.).

For the D10-PfCyc1-DD parasites, the D10 strain of *P. falciparum* (Walter and Eliza Hall Institute) was transfected with the targeting plasmid pJR13, maintained on 0.25 μM Shld, and cycled on and off WR99210 selection to obtain parasites with a stable single crossover. Individual transgenic parasites were obtained by limiting dilutions. For live parasite nuclear counting, parasites were stained in a Hoechst 33342 solution of 1 µg/ml for 10 min, allowed 10 min to adhere to 0.5 mg/ml concanavalin A-coated microscopy dishes (Ibidi), and then complete medium was added. Parasites were imaged with serial sections on an inverted Nikon Eclipse Ti wide-field microscope with a 60× 1.4NA objective.

### (v) Yeast two-hybrid assay.

Strain PJ69-4α was transformed with indicated pGBKT7 backbone bait plasmids, and strain PJ69-4a was transformed with indicated pGADT7 backbone prey plasmids by standard methods (pGBKT7 and pGADT7 from Takara Bio USA) (29). Bait and prey strains were mated, and diploids were selected on synthetic media lacking leucine and tryptophan. Bait-prey interaction was tested in diploids by dilution plates onto media lacking leucine, tryptophan, histidine, and adenine. The ability to grow in the absence of histidine and adenine requires interaction of bait and prey hybrid proteins to drive transcription at two distinct GAL4-responsive loci.
